# High-Performance, Low-Cost, Additively Manufactured Electrospray Ion Sources for Mass Spectrometry

**DOI:** 10.1021/jasms.3c00409

**Published:** 2024-03-22

**Authors:** Alex Kachkine, Luis Fernando Velásquez-García

**Affiliations:** a Department of Mechanical Engineering, Massachusetts Institute of Technology, 77 Massachusetts Avenue, Cambridge, Massachusetts 02139, United States; b Microsystems Technology Laboratories, Massachusetts Institute of Technology, 77 Massachusetts Avenue, Cambridge, Massachusetts 02139, United States

## Abstract

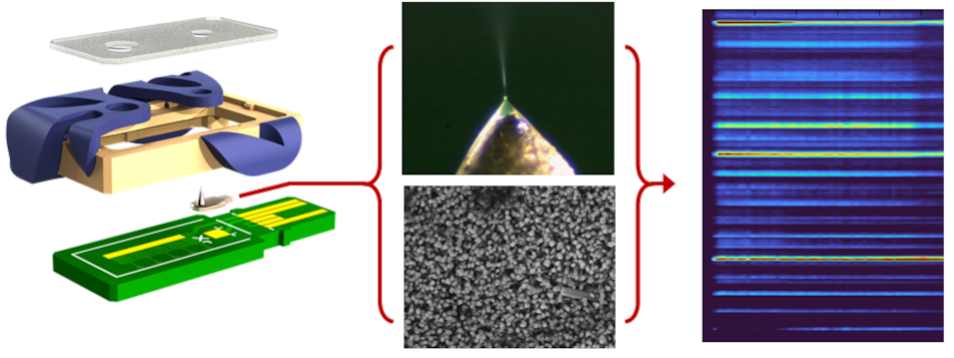

We report novel 3D-printed electrospray sources for mass spectrometry (MS) that produce twice the signal strength of their mainstream counterparts. Leveraging 3D printing to fabricate in bulk nano- and microscale-featured electrospray emitters, this work shows a path for scalable integration in clinically relevant diagnostics. This solution improves the device performance by simultaneously tuning the surface hydrophilicity, solvent evaporation, and geometry. The emitters are made of stainless-steel (SS) 316L via binder jetting and coated in a conformal, hydrothermally grown zinc oxide nanowire (ZnONW) forest. The printed emitters are designed as surface mount devices that can be directly soldered to printed circuit boards with built-in digital microfluidics as part of an automated device assembly. The electrospray sources use a novel extractor electrode design that enables operation at ∼24% larger bias voltages compared with conventional MS cylindrical inlets. The 3D-printed electrospray emitters were characterized against their state-of-the-art counterparts (coated blades and paper spray). MS data from the 3D-printed electrospray emitters show detection of therapeutically relevant targets at 1 μg/ml concentrations with a variety of solvents; for nicardipine, such emitters attain 116% higher signal-to-noise ratios and far greater stability than their counterparts.

## Introduction

Clinical MS of biological liquids, e.g., serum, allows for fast and sensitive analysis of biological analytes. While MS can provide multiplexed information, it has yet to reach its full clinical potential.^1^ MS applications have been hindered by difficulties in constructing robust sample processing and ionization workflows, with existing methods requiring significant manual operation.^2^ From crude samples, liquids undergo target extraction prior to ionization to minimize background noise and concentrate the target.^3^ Common methods to do so span solid-phase extraction,^4^ matrix-assisted elution,^5^ liquid chromatography,^1^ electrophoresis,^6^ and phase extraction via slug flow.^7^ Signal strength derives from the number of target ions entering the MS instrument, with several confounding factors; poor ionization can cause noise-inducing molecular fragmentation, low repeatability, and loss of recorded intensity.^8^ Such issues have spawned a library of counter-techniques that benefit from having an effective ion source; consequently, many reported ionizer designs aim to attain high current and homogeneous ionization, ideally leading to a stream of singly ionized molecules.^9^

Ionization of liquids is commonly done via electrospray, i.e., the electrohydrodynamic jetting of charged particles from a liquid surface subjected to a high electric field.^9^ Assuming steady conditions and an applied electric field above a certain threshold on the liquid free surface, a stable Taylor cone^10^ forms that emits charged droplets from its tip. Stability regimes of electrospray emitters with conical geometries have been characterized for well-controlled capillary flow, with different operating parameters leading to intermittent ion jetting, chaotic behavior, or stable cone-jet emission.^11^ With sufficient starting charge, individual molecules can be isolated via electrospray, reducing the noise in MS measurements. Ions can also be directly emitted from a liquid surface, i.e., field evaporated.^12^ Pure ion emission from ionic liquids likely undergoing field evaporation in vacuum-operated ZnONW-coated emitters has been reported.^13^ To the best of our knowledge there are no reports of electrospray sources attaining atmospheric pressure field evaporation of organic solvents, although corona discharge effects in paper electrospray suggest the possibility.^14^ Hardware development including nanocapillaries, microfabricated emitter arrays, and externally wetted emitters have reportedly improved ionization efficiency, defined by the charge-to-molecule ratio in an emitted jet.^15,16^

The morphology of an electrospray emitter influences its performance. Internally fed emitters, i.e., capillaries,^17^ suffer from a variety of issues ranging from clogging to difficulty of integration. Furthermore, cleanroom microfabricated versions^18^ are expensive and time-consuming to make. There is a large volume of research on low-cost liquid ionizers such as paper spray.^19^ Unfortunately, such devices are often difficult to integrate into MS protocols; for example, cartridges for paper spray increase the cost of the ionizer to levels comparable with capillary sources,^20^ and they are hard to manufacture due to the handling difficulties associated with paper, particularly at the microscale. Vibrating edge nebulization ionizers, though simple electrically, have poor signal properties.^21^ Coated blades have recently emerged as electrospray ion sources for MS that balance cost with performance.^22^ We recently demonstrated solid cones coated with a hydrophilic ZnONW forest that attain higher peak ion currents than their counterparts, with sharper tips and conductive substrates plausibly playing a role in ion jet behavior.^23^

The shape of the extraction electrode also plays an important role. Some extractor designs leverage Coandă flow, e.g., ref (24), to maximize signal intensity. Others collimate desolvated ions into the MS instrument inlet, e.g., ref (25). The dynamics of electrospray are significant beyond ion emission; variability in the direction of an electrospray jet, radial spread from space charge effects, and positioning all significantly affect MS signals.^26^ These effects can lead to counter-intuitive results, where lower electrospray currents have higher MS signals, with the stability of Taylor cones reducing beam divergence.^27^

Methods for interfacing sample processing with liquid ionization span in-line approaches with syringe-based matrix filtration,^28^ electrophoretic separation of target analytes,^29^ and intracapillary methods.^30^ Digital microfluidics (DMF) is a proven technology in a range of biotechnology uses, where droplets are moved by applying voltages to electrodes coated with a dielectric and hydrophobic layer.^31^ DMF devices can be made with low-cost, flame-retardant printed circuit boards (FR4 PCBs).^32^ Because electrospray ionization requires a high bias voltage, the integration of additional circuitry for DMF control is marginal, making the technique attractive for consumable development. Target extraction on DMF devices has been demonstrated with solid-phase extraction,^33^ while MS integration has been accomplished using horizontal capillaries and vertical orifices.^17^ These coupling methods for electrospray have yet to be widely adopted owing to the difficulty in fabricating and positioning the devices. Recent advancements in additive manufacturing, e.g., voxel miniaturization, have enabled the direct printing of arrays of internally fed electrospray emitters,^34^ microfluidic chips,^35^ and desorption electrospray platforms.^36^ While 3D printing is still costly compared to mass-manufacturing techniques such as injection molding,^37^ the limitations of the latter have pushed the industry toward additive approaches.^36^

This study focuses on the ionization step in standard MS protocols, concentrating on combining mainstream 3D printing methods and nanostructured surface treatments to achieve scalable manufacturing of high-precision hardware with automatable assembly. Leveraging the reported good performance of 3D-printed, externally fed, ZnONW-coated emitters in a vacuum,^13^ we model their operation at atmospheric pressure and subsequently optimize their morphology to attain high-current emission of homogenously ionized particles. We design extractor electrodes for maintaining adequate electric fields at the surface of the emitter without causing air breakdown. Optimized devices are placed in world-to-chip style interfaces and characterized, showing that the 3D-printed emitters surpass current mainstream approaches for point-of-care electrospray ionization.

## Experimental Section

### Electrospray Signal Maximization

Electrospray ion emission was modeled to account for evaporation and geometric variation, which is especially relevant for volatile solvents with surrounding gas flow, such as in MS setups. Via first-principles reduced-order modeling, the startup criteria for electrospray regimes, their relation to emitter geometry, the expected currents, and the ion properties are connected to the fluidic properties and manufacturing tolerance of externally fed emitters as well as to evaporation phenomena. The effects of the extractor electrode geometry on air breakdown are also analyzed. A high-level description of the modeling is provided below; further details are provided in the Supporting Information.

#### Emitter Electric Field

The electric field at the tip of a hyperbolic liquid surface (eq S1) triggers electrospray at a threshold field strength (eq S2).^16^ Going past this value, the field evaporation threshold field strength (eq S3) is eventually reached, whereby ions are directly emitted.^12^ Operational values fall between these two bounds (Figure S1).

#### Emitter Ionization Currents

Once electrospray has been initiated, the electrospray current (eq S4) is driven by the flow rate and properties of the liquid.^38^ Higher MS signal intensity results from reducing the flow rate, a consequence of molecules having higher charge, increasing current-carrying capacity.

#### Emitter Evaporative Effects

Evaporation is approached as a regulation scheme to constrain flow and ensure signal stability. Externally fed electrospray emitters are modeled as solid cones (Figure S2). Their surface flow rate (eq S6) is approximated as that of a revolved array of cylindrical capillaries scaled-down per conical surface area,^39^ with Young–Laplace pressure driving the flow.

In atmospheric pressure electrospray, air flow significantly affects evaporation rates, invalidating traditional approaches for modeling the evaporation rate of electrospray in a vacuum. Advancements in other fields have led to empirically accurate evaporation rate predictions that are dependent on the velocity of the surrounding fluid.^40^ Air in the inlet of an MS instrument moves close to the speed of sound, owing to the internal vacuum, with airflow near the inlet flowing at several meters per second.^41^ Volatile organic solvents commonly used in MS electrospray evaporate quickly at small scales.^42^ The model applies the Hummel–Braun–Fahrenbacher evaporation rate,^40^ which is scaled to the surface of a conical emitter (eq S7).

#### Emitter Manufacturing Tolerance

Geometric variations from additive manufacturing are larger than those from semiconductor cleanroom processing.^13,16^ Wider emitter tip angles allow for greater robustness to these errors, although they also conflict with flow rate optimization. This may be addressed with more precise printing methods.

#### Extractor Electric Field

The primary physical constraint on the maximum bias voltage that can be applied to an emitter at atmospheric pressure is air breakdown (around 3 V/μm^43^), tied with the formation of a continuous plasma channel between the tip and the extractor electrode. A sharp emitter can easily trigger air breakdown; therefore, instead of limiting the tip field strength, this study aims to reduce the plausibility of creating a continuous channel of plasma between the two electrodes, i.e., by avoiding a continuous, high-electric-field path connecting regions of both electrodes. The distance between the emitter and the extractor is reduced to decrease space charge effects, e.g., divergence and the subsequent loss of emitted ions. Three extractor designs (design A, a sharp cylindrical electrode; design B, a smoothed electrode; and design C, a smoothed electrode with internal hyperbolic curvature) were characterized, as detailed in the Results. The optimal design was found to be C, which was used in the MS experiments.

### Ionizer Device Design

Two ionizer designs were explored in this study. The first is a DMF chip with a surface mounted emitter, a consumable form factor that demonstrates the scalable architecture of additively manufactured MS sample preparation hardware. The second is a planar ionizer with a horizontally oriented emitter at its end, which is made for streamlined emitter evaluation with a commercial compact MS instrument.

#### Ionizer with Surface Mounted Emitter

**Figure 1:**
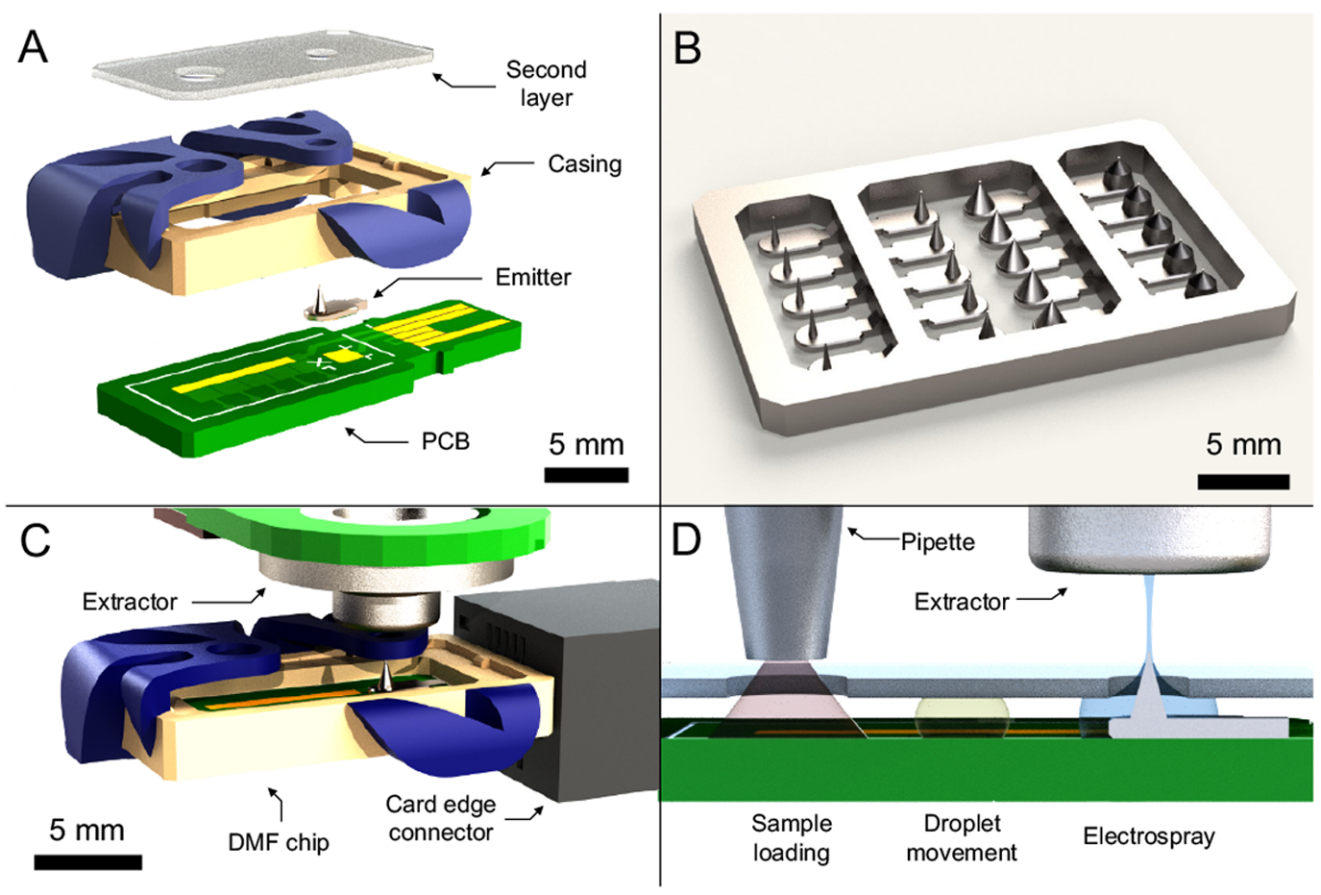
Selected rendered CAD views of the ionizer with a surface-mounted emitter. (A) Detailed view of the device; the two cropped contact pads of the card edge connector are shorted and serve as position verification electrodes. (B) A 3D-printed array of tethered surface mount emitters spanning a range of tip radii, surrounded by a handling frame that protects the emitters during batch processing. (C) Ionizer integrated to an experimental apparatus via a card edge connector and a separate extractor. (D) Exemplary workflow shown in cross-section view: a sample droplet is loaded by a micropipette, and the droplet undergoes on-chip manipulation, eventually being fed and ionized by the vertically oriented electrospray emitter.

Rendered computer-aided design (CAD) views of the ionizer with a surface mounted electrospray emitter are shown in Figure 1. Each device comprises a DMF PCB, a soldered 3D-printed emitter, and an external 3D-printed casing. Using the DMF PCB, droplets can be moved around as part of solid-phase or liquid extraction protocols^17^ prior to movement onto the soldered electrospray emitter. The external casing positions the internal components of the ionizer, enabling easier handling and usage. The ionizer is controlled and positioned via a card edge connector. This form factor is mass-producible: all components are laterally positioned and vertically assembled. Relevant to long-term applications, the vertical alignment of the emitter and extractor requires a compatible vertically oriented MS inlet, which is not found in commercial compact MS systems.

#### Planar Ionizer

**Figure 2:**
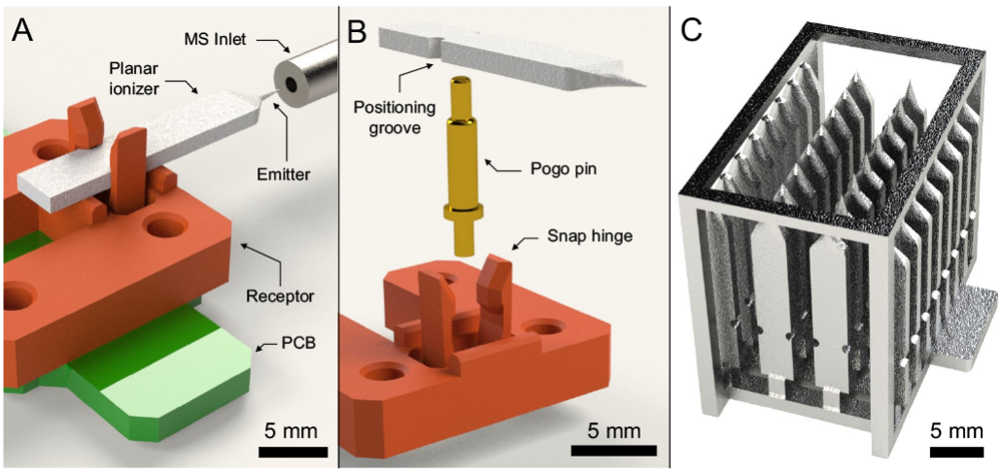
Rendered CAD views of a planar ionizer with a horizontally mounted emitter. (A) Assembly showing the relative positioning of functional components in an MS testing protocol. Note that a custom extractor (not shown) was mounted onto the conventional inlet for experimentation. (B) Focused view showing the planar ionizer and receptor. (C) 3D-printed array of planar ionizers surrounded by a cage that protects the emitters during batch processing.

To streamline emitter characterization and ensure compatibility with conventional horizontally oriented inlets of the MS instrument used in this study, we also integrated the 3D-printed emitters in a planar configuration (Figure 2). In this format, the emitter is horizontally appended to a planar substrate. The planar form factor allows direct comparison of the performance of the 3D printed emitters with their mainstream counterparts (paper spray and coated blades). This form factor enables direct deposition of a neat sample with immediate migration to the emitter. The 3D-printed planar ionizers are compatible with bulk fabrication (Figure 2C): arrays of aligned ionizers can be processed to apply necessary surface coatings to the emitter, which are extended to the entire surface to enable capillary surface-driven flow toward the emitter.

### Ionizer Fabrication

A high-level description of the fabrication of the devices is provided below; further details are given in the Supporting Information. The ZnONW coating protocol was adapted and iterated upon from ref (13).

#### Emitters

Two versions of 3D-printed emitters were explored (Table S1). The emitters were made in SS 316L via binder jetting (i.materialize, Leuve, Belgium) and coated in hydrothermally grown ZnONWs. Emitter variant *E*_1_ was electropolished. The emitter height was set at 1.5 mm, and the emitter tip was equal to 15°, 30°, 60°, or 98.6° (i.e., the Taylor cone angle).

#### Extractor Electrode

The extractors were made in SS 316L via binder jetting (i.materialize, Leuven, Belgium). The extractors were mounted on PCBs with edge card connectors.

#### Ionizer with Surface-Mounted Emitter

**Figure 3:**
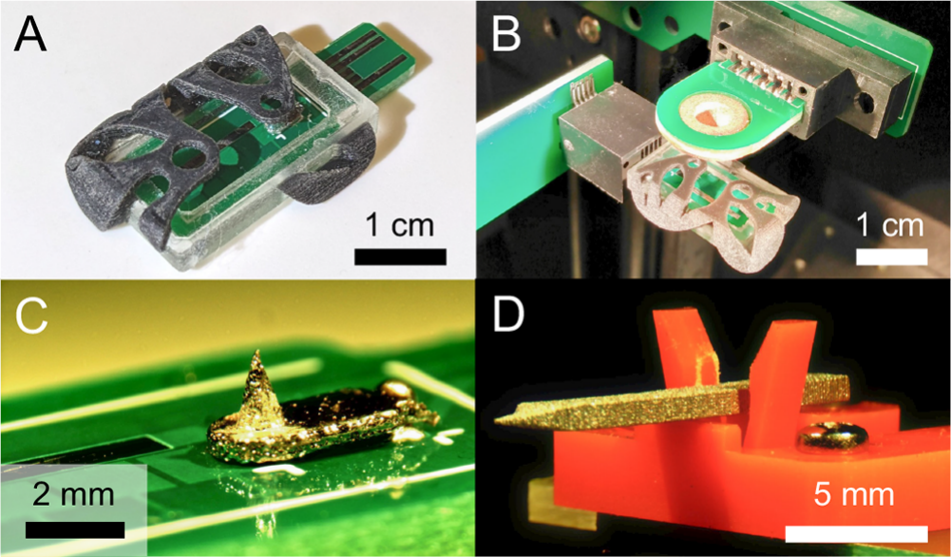
Selected images of the MS ionizers developed in this study. (A) Fully assembled ionizer with a surface mount emitter. (B) Ionizer with the surface mount emitter inserted in a receptor with a vertically aligned extractor positioned above the emitter. (C) Surface-mounted emitter *E*_1_ soldered to a DMF PCB. (D) Fully assembled ionizer with a planar *E*_2_ emitter.

The surface mount emitter is reflow soldered onto the DMF PCB, and all components are snap-fit into the external casing. A layer of silicone adhesive was applied to fully secure the components to the casing. The electrospray emitters are integrated into the ionizers via pick-and-place methods used in commercial PCB production, taking advantage of the fact that the 3D-printed emitters have sufficient contact area for vacuum-based pick-and-place instrumentation to operate. Soldering temperatures do not affect the ZnONW coating, as the crystal forest is stable at temperatures above 1000 °C.^44^ Images of assembled ionizers with a surface-mounted emitter are shown in Figures 3A–C.

#### Planar Ionizer

The receptors used in planar ionizers were printed via vat photopolymerization in SolusProto resin (Junction3D, Santa Clarita, Ca, USA) using an Asiga MAX X27 instrument (Asiga, Alexandria, Australia). To avoid sample leakage onto the receptor, a Teflon AF 2400X (DuPont, Wilmington, USA) dip-coat is performed on the rear of the ionizers. The 3D-printed planar ionizer is snapped into the 3D-printed receptor on a PCB with an underlying pogo pin for electrical actuation. An assembled planar ionizer is shown in Figure 3D.

### Experimental Characterization

Experiments were conducted to characterize emitter metrology, extractor electrical performance, electrospray dynamics, droplet movement, and MS spectra. A high-level description of the characterization is provided below; further details are supplied in the Supporting Information.

#### Emitter Metrology

The bulk geometry of fabricated emitters was characterized using a Moticam S20 camera (MOTIC USA, Schertz, TX, USA), an AmScope MT5000-IFR camera (AmScope, Irvine, CA, USA), and a Zeiss Gemini 450 scanning electron microscope (SEM) (Carl Zeiss AG, Oberkochen, Germany). Image scale calibration and subsequent measurements were conducted in ImageJ (US National Institute of Health, Bethesda, MD, USA). Emitters were imaged laterally for a side-profile view, with the mean and standard deviation collected for tip angle, bulk tip radius, and emitter height. The bulk tip radius was determined by fitting a circular arc to the bulk shape of the emitter.

#### Extractor Performance

Triplicate voltage sweeps were conducted to identify the level at which air breakdown (arcing) occurred. Extractors were positioned such that their front plane was offset by 1 mm from the tip of emitter *E*_2_ with a 60° tip angle. Positive DC voltages were applied to the emitter relative to the grounded extractor with a Keithley 2657a source-measuring unit (SMU) (Tektronix, Beaverton, OR, USA) in 50 V increments before breakdown occurred.

#### Droplet Movement

Fabricated DMF devices with surface-mounted emitters were evaluated for basic performance. A device with a soldered surface mount emitter was inserted into a card edge receptor, with vertically oriented extractor design C positioned 1 mm above the emitter tip. A 5 μL drop of 95% water and 5% acetic acid was deposited on the device and manipulated via actuation of underlying square electrodes with a Keithley 2657a SMU instrument. Following drop transfer onto the emitter, electrospray was initiated by positively biasing the emitter relative to the grounded extractor.

#### Electrospray Dynamics

Emitters were characterized optically and electrically to understand modeling relevance, especially the effect of surface flow regulation on electrospray behavior. High-framerate, high-resolution imaging was conducted with a Moticam S20 camera (AmScope, Irvine, CA, USA) interfaced with a 3X camera tube, a 7X zoom module, and a motorized 4X lower lens (Edmund Optics, Barrington, NJ, USA). A 20 W LED illumination source (Amscope, Irvine, CA, USA) from the front and rear of the experimental setup enabled visualization of plume dynamics with good precision (sub-micrometer pixel sizes at 50 frames per second with 5-megapixel images). To enable fast iteration of experimental parameters, planar ionizers with different emitter types were snapped into the bespoke receptor. Voltages were supplied by a set of Keithley 2657a SMU instruments. Extractor design C was positioned 1 mm away from the emitter tip and grounded, and a DC positive bias voltage was applied to the ionizer in a single step via the pogo pin in the receptor. A 5 μL drop of isopropanol was applied to each ionizer before biasing the voltage to trigger electrospray, with time-series data and sweep data collected at a rate of ∼10 Hz. Simultaneous optical data collection was conducted. Peak steady-state currents were identified from time-series data, while incremental voltage sweeps gave maximum operating voltages, all taken as the average of at least three experiments. Due to the imprecision of defining steady-state and variable breakdown conditions with solvent presence, 25 V increments for steady-state current and 250 V increments for maximum operating voltages were used when reporting measurements.

#### MS Spectra

Planar ionizers with horizontally mounted emitters were used for a proof-of-concept evaluation of the suitability of the emitters developed in this study in MS protocols. For these experiments, a BaySpec Continuity transportable MS instrument (BaySpec, Inc., San Jose, CA, USA) was employed in ESI mode alongside a Keithley 2600B power supply (Tektronix, Beaverton, OR, USA) for electrical connections to the MS inlet and emitter. The MS has a sharp, horizontally oriented, ∼2 mm diameter inlet with a 500 μm diameter orifice. Given that this extractor configuration limits the performance of electrospray emitters due to air breakdown, extractor C was attached concentrically to the end of the inlet during the experiments. MS data were collected at the maximum voltage (experimentally determined) at an inlet distance of 2 mm. Planar ionizers with emitter types *E*_1_ and *E*_2_ were coaxially positioned on the grounded MS instrument orifice. A 10 μL droplet of sample was dropped onto the ionizer and subsequently energized, producing electrospray. Data collection occurred continuously, with the instrument recording spectra with 1 *m*/*z* resolution at ∼1 Hz. The sample comprised ten pharmaceutically relevant compounds spiked in serum at a concentration of 1 μg/ml in either isopropanol, methanol, or acetonitrile. The target compounds were lisinopril, enalapril, clopidogrel, nicardipine, apixaban, atorvastatin, rosuvastatin, pravastatin, aspirin, and rivaroxaban. Time-series data were aggregated into composite mass spectra before peak identification with the find peaks method of SciPy,^45^ filtering for peaks with a minimum average signal intensity of 5000 with a distance parameter of 1. The resulting peaks were matched to expected peaks in the literature, though with limited accuracy owing to instrument-related signal shifting. For the clearly identified nicardipine, signal-to-noise ratios were calculated for comparison to those of coated blades. We also obtained data using the default capillary electrospray source of the MS instrument for a rough signal comparison. Additionally, paper spray did not produce an identifiable signal for nicardipine in the experimental protocol. To allow for rough MS data comparison, we obtained a nicardipine signal for paper spray by relaxing the method parameters: the sample was just nicardipine spiked in methanol at 1 μg/ml (no other compounds were added), and the signal threshold intensity was reduced from 5000 to 50.

## Results

### Evaporative Modeling

**Figure 4:**
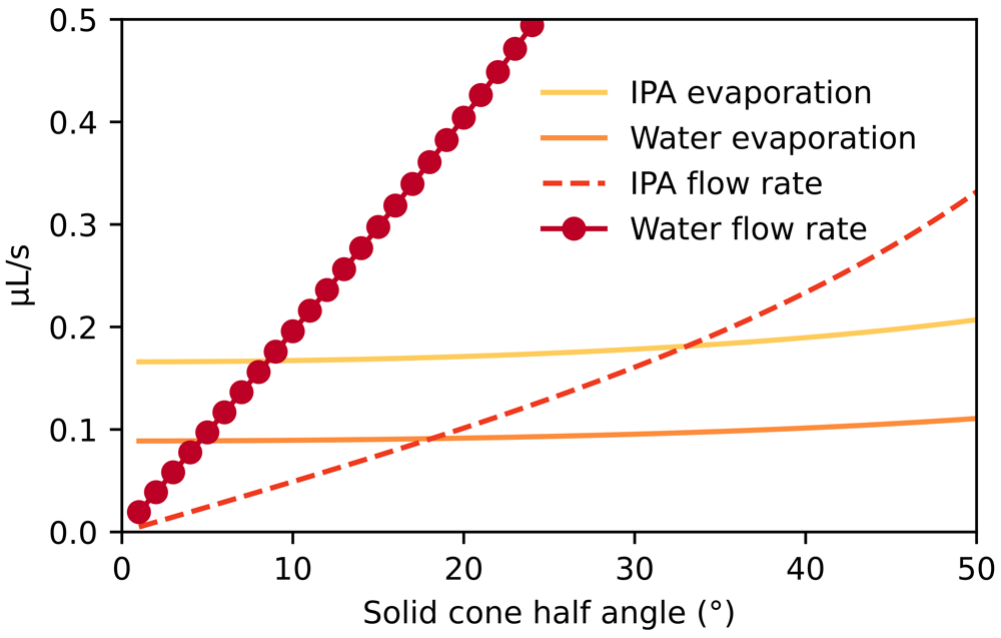
Evaporation and surface flow rates for water and isopropanol on a 1 mm tall ZnONW-coated electrospray emitter. For 2-propanol, the larger vapor pressure leads to a larger required cone half angle (∼33°, marked by blue arrow) for solvent to reach the tip and initiate electrospray. For water, shallower emitter tip half angles down to ∼5° can be tolerated with a positive flow to the emitter tip. At small tip angles, the evaporation rate converges to a positive value despite the shrinking surface area; this is a consequence of the limit of molecular diffusion into surrounding air when the amount of unsaturated air is vast compared to the area of the fluid interface, which is inaccurate as angles approach 0°.

Figure 4 shows how the surface and evaporation flow rates vary for water and 2-propanol for an atmospheric pressure MS setup. Evaporation sets a lower bound on the tip angle that can support solvent flow for electrospray to occur. In other words, the surface flow rate can be tuned by setting the tip angle and surface porosity. A minimal flow rate can be targeted, achieving higher ionization efficiencies. This is a remarkably straightforward approach, and to our knowledge this is the first study to quantitatively model the approach. The interactions between surface flow and evaporation may explain the performance of many conventional electrospray sources, for instance, directly modeling the results of ref (42) where an evaporation barrier improved paper spray stability and longevity. Higher air flow rates near an MS inlet can cease electrospray altogether, an important barrier hitherto unquantified in electrospray source design.

### Extractor Modeling

**Figure 5:**
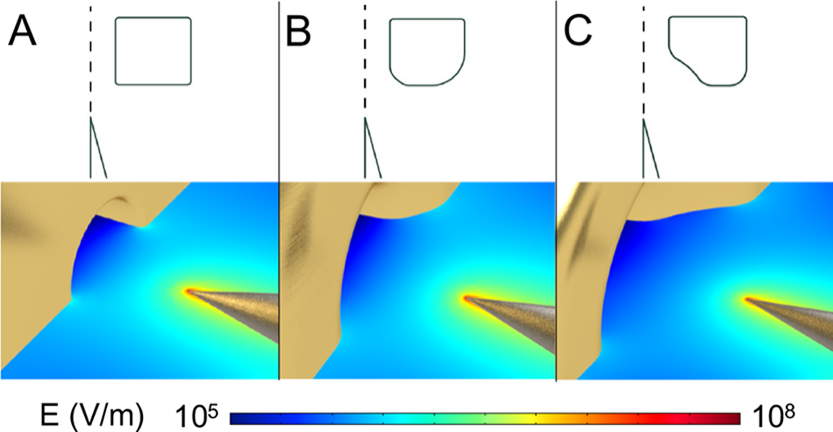
Electric field strength simulation results of emitter–extractor electrode diodes. The same emitter geometry and nominal emitter-to-extractor separation is used. Revolved profiles of the emitter and extractor are on the top of each panel, while model renderings overlaid on simulated electric field cross-sections are shown at the bottom of each panel. In each case, a grounded extractor with a 1.5 mm diameter orifice was positioned 1 mm away from the tip of a conical 30° tip angle emitter with a 15 μm tip radius biased at 3 kV. (A) Results using extractor design A. (B) Results with extractor design B. (C) Results with extractor design C. The maximum surface field strength of the extractors decreases when going from designs A to C, with design C not having a continuous high electric field region between the emitter and the extractor capable of creating an arc.

Finite element simulation results show that the maximum surface field strength on the emitter is always far larger than the air breakdown threshold (Figure 5). In the case of the sharp, cylindrical extractor A, there are continuous regions of high field strength (>3 V/μm) connecting the extractor and emitter surfaces and inducing arcing. Smoothed extractors B and C do not have such continuing high field strength paths, thus avoiding connected air ionization regions while maintaining roughly the same field strength on the emitter tip.

### Extractor Performance

For the standard cylindrical extractor (design A), electrical runoff occurs with an applied bias voltage of 2.5 kV at an emitter–extractor distance of 1 mm while using emitter *E*_2_ as a counter-electrode. Imaging of the arc discharge between the emitter and the extractor (Figure S3) shows that the arc forms from the tip of the emitter to the inner edge of the extractor; this is in accordance with the electric field distribution shown in Figure 5 and evidences the shape optimization that must be conducted to limit electrical discharge, which otherwise compromises the tip of the emitter by burning off surface features. Using extractor B, the same kind of emitter, and same emitter–extractor separation, the bias voltage required to cause air breakdown increased to 2.8 kV. Similarly, using extractor C, the bias voltage required to cause air breakdown increased to 3.1 kV. Such an increase in the maximum bias voltage is significant to the optimization of signal strength, as larger applied bias voltages lead to more homogeneous ionization of single particles. Consequently, the electrospray characterization experiments were conducted using the extractor design C.

### Emitter Metrology

Metrology of the 3D-printed electrospray emitter designs in this study is summarized in Table S2. Sharpened metal emitters *E*_1_ had the most accurate fabricated tip angles (0.9° error) and the smallest tip radii (56.0 μm). Unsharpened metal emitter *E*_2_ had the most accurate fabricated tip height (24 μm error). SEM pictures of the fabricated emitters are shown in Figure S4. ZnONW forest homogeneity is reported in the Supporting Information.

### Electrospray Dynamics

**Figure 6:**
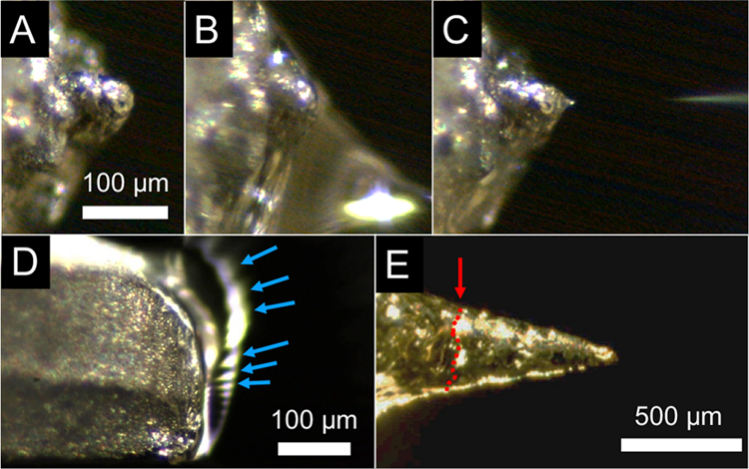
Selected time-series screenshots showing differences in emitter dynamics due to surface treatment and emitter geometry. (A–C) Documented progression from wetting to electrospray for an *E*_2_ emitter at 3 kV and a 98.6° tip angle; panels A–C share the same scale bar. (D) Coated blade with electrospray in the steady state, showing rapid instability of the electrospray along the spatially large tip region; arrows mark the observed locations of jets within the exposure period of the photograph (20 ms). (E) *E*_1_ emitter with a 15° tip angle; the arrow and dotted line mark the extent to which isopropanol was able to crawl on the emitter before evaporating.

Figure 6 shows representative time-series images of several emitter designs during operation, evidencing the effects of emitter geometry and surface roughness on solvent flow, jet formation, and jet stability. Paper spray and coated blades had wide variability in electrospray behavior and evidently lower electrospray efficiency, as determined by bulk jet formation and a lack of immediate plume divergence. With coated blades, the metal tip is not hydrophilic, and a highly unstable electrospray jet forms on the large tip region. The optical data show that tip angles and surface coatings significantly affect the solvent reaching the tip of emitters. Table S3 summarizes the experimental observations, while typical time-series and voltage sweep current measurements are plotted in Figure S5, with corresponding discussion of individual emitter behavior in the Supporting Information. Repeated voltage sweeps without replenishing the liquid show qualitatively the same behavior, i.e., convergence around a steady-state current after an initial burst. The initial burst is attributed to a larger drop of solvent that is first rapidly electrostatically drawn toward the tip of the emitter (the voltage is not ramped up but applied as a step function) to form a bulk Taylor cone. Within a few seconds, evaporative effects constrain the flow to steady-state electric traction and a hydrophilic-driven surface flow behavior. Electrospray startup voltages did not substantially vary, generally falling in the 2–2.25 kV range. We attribute this result to the bulk formation behavior present for all tested emitter types; the large amounts of fluid electrostatically pulled onto the emitter from the initial applied voltage behave the same regardless of the microscale tip features.

### MS Characterization

#### Solvent Compatibility

**Figure 7:**
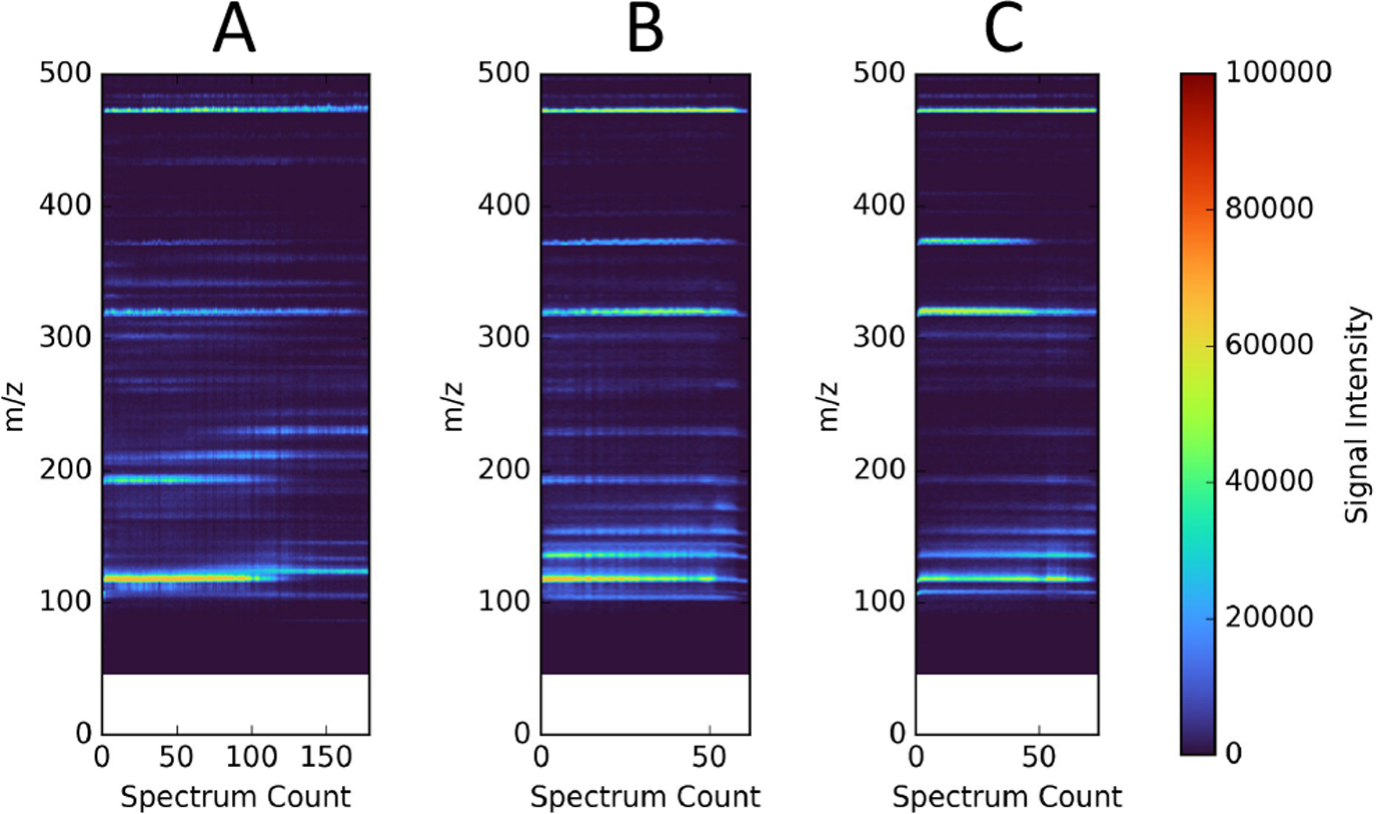
Time-series MS data for samples ionized by a ZnONW-coated *E*_1_ emitter using (A) isopropanol, (B) methanol, and (C) acetonitrile as the solvent. The data show the stability of the signal intensity (common axis at right). The shifting signals are due to the MS instrument, as confirmed by the manufacturer. The spectrum count corresponds to time with roughly 0.7 s per spectrum. The extended duration of the 2-propanol spectra is attributed to the greater stability of the electrospray and the lower vapor pressure of the solvent.

Time series data are shown in Figure 7 for all three solvents tested, evidencing the homogeneity of the signal intensity. The data show compatibility with multiple solvent types for ZnONW-coated emitters, excellent signal duration, and good signal intensity for diagnostic applications. Derived aggregate MS spectra are plotted in Figure S6, with the most prominent peaks labeled by their charge-to-mass ratio, and five target compounds identified.

#### Comparative Testing

**Figure 8:**
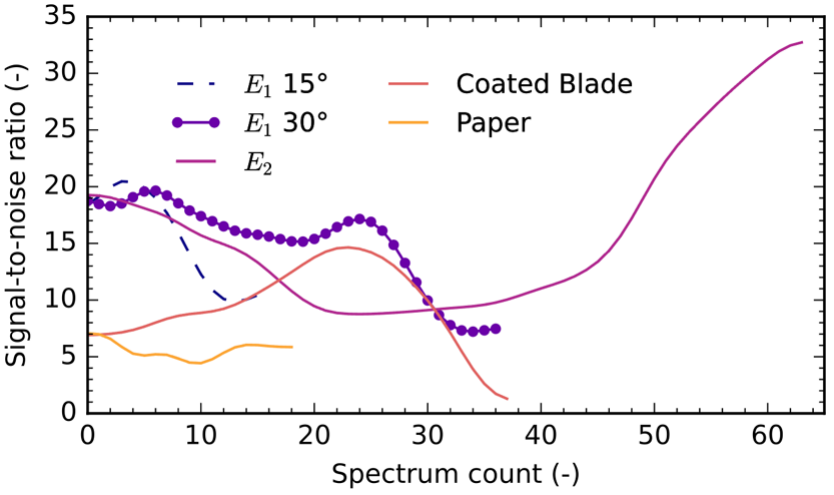
Signal-to-noise ratio vs time (spectrum count) for nicardipine using five types of emitters. For ease of interpretation, data were smoothed with a Gaussian filter (σ = 2). ZnONW-coated emitters outperform their counterparts in terms of peak signal-to-noise ratio and duration at peak. *E*_2_, at a solid cone half angle of 15°, has a peak near the end of data collection as the solvent runs out and high efficiency ionization is reached. The different solid cone half angles of *E*_1_ evidence a tradeoff in peak intensity and signal duration: a shallower tip exhausts solvent quicker but reaches a higher peak signal. Paper spray, even with relaxed method constraints, shows a peak signal substantially below that of other emitter types.

MS data from two ZnONW-coated emitters and a coated blade are shown in Figure S7. The derived time-series signal-to-noise ratio for nicardipine is plotted in Figure 8, and it is highest for ZnONW-coated emitters; this high intensity is short-lived for emitter *E*_1_, but it is long-lasting for emitter *E*_2_, which also displays a terminal peak, possibly indicating a transition to higher ionization efficiency. Relative to the coated blade, *E*_1_ had a peak signal-to-noise ratio 65.3% higher, while that of *E*_2_ was 116% higher. Compared to the default capillary electrospray source, the peak signal-to-noise ratio for *E*_2_ was 39.3% higher. Although it is significant that the 3D-printed ionizers’ performance is comparable with that of the capillary ionizer, it is important to point out that direct comparison between externally fed and capillary ion sources is misleading owing to fundamental differences in experimental setup and sample size. Capillary ionizers involve fixtures, voltage sources, and fluidic pumps that are not employed when running externally fed ionizers. Further, capillary emitters run with a continuous flow rate, while externally fed emitters work with a fixed sample volume. While paper spray did not show a clear nicardipine signal using the original protocol, relaxing the constraints allowed signal identification at a peak signal-to-noise ratio 42.4% of *E*_2_.

### Droplet Movement

Droplet movement across the DMF PCB is achieved with DC bias voltages as low as 250 V, although reliable operation was attained with 600 V. Using the DMF PCB, droplets could readily be transferred onto the soldered emitter to be electrosprayed (Figure S8).

## Discussion

This study targeted the development of low-cost electrospray sources for point-of-care (PoC) mass spectrometry by harnessing additive manufacturing to batch fabricate finely structured, millimeter-scale parts with precise geometry at scale. While developing atmospheric pressure electrospray emitters with high-performance ZnONW coatings, the geometry of the extractor electrode was optimized to reduce air breakdown; the optimized extractor allows for operation at 24% higher voltages before reaching breakdown, improving the robustness of the ionization protocols.

To the best of our knowledge, this study is the first effort to analytically explain evaporation-inflicted limits on externally fed electrospray emitter performance; experiments confirm that wider tip angles are necessary for volatile solvents on low-porosity surface coatings. Larger MS signals can be achieved by strategically restricting solvent flow via evaporation, a matter partly explained by the higher charge-carrying capacity of ions versus neutral molecules in a jet.

Characterization of the 3D-printed electrospray ion sources with a portable commercial MS instrument shows good solvent compatibility and identification of clinically relevant pharmaceutical compounds. Focusing on the clearly identifiable nicardipine peak, the data show that the 3D-printed emitters achieve up to 116% higher signal-to-noise ratios compared to those of state-of-the-art coated blades. The signal-to-noise ratio has a time dependence affected by the tip angle of the emitter, with shallower emitters attaining higher peaks for shorter durations. This behavior is related to solvent evaporation, though some signal improvements may derive from ion focusing, which has been simulated with internally smoothed extractor designs.^46^

Relevant to long-term applications, the duration and signal stability of ZnONW-coated, binder jetting-printed metal emitters are unparalleled compared to the mainstream PoC emitters studied in this work (paper spray); this suggests broad applicability of the technology in PoC settings. We step forward toward this goal by integrating the emitters in a first ever solderable surface mount form factor on a PCB with a DMF device for sample processing. We demonstrated droplet loading, movement, transfer onto the emitter, and subsequent electrospray into a vertically oriented extractor. Handling, positioning, and control interfaces are readily implemented to reduce operational constraints.

Optical imaging of Taylor cones from the 3D-printed emitters confirms that topological optimization strategies driven by evaporative loss ensure robust operation of the externally fed emitters. Although the literature treats solvent evaporation outside of electrospray ionization as a burden, we approach the phenomenon as a tool for engineering steady-state behavior and obtaining higher ionization efficiency. Absent such optimization, bulk fluid behavior pervades for large tip angles, resulting in an equivalent performance across emitter types. We suspect that many externally fed sources reported in the literature, such as paper spray, experience the same bulk-fluid-driven performance limitations and are thereby equivalent; ionizer improvements are likely tied to other hardware changes. While evaporative tuning enables optimal emitter design, the resulting emitter geometry relies on the properties of a specific solvent and surrounding conditions such as air temperature; therefore, the performance of optimal designs when they are used in different protocols should be affected.

The solderable emitter paradigm presented in this study could slash the labor costs of ionizer assembly. It is tempting to ascribe the lowest cost to methods with the lowest material value, e.g., paper spray; these conclusions forego the discussion of integration, which is often prohibitively difficult in automated production owing to inhomogeneous material properties or handling requirements. For instance, while direct integration of paper spray into a DMF device printed on paper^47^ can be scaled, paper is difficult to handle and interface with external electronics, requiring cartridges^20^ that undo the advantages of low-cost materials. While the costs of electropolishing, chemical processing, and storage incurred by our emitters are significant, this study has introduced a potentially game-changing paradigm from an assembly standpoint: our emitters can be handled and soldered like any surface-mounted component in conventional circuitry using pick-and-place hardware. To the best of our knowledge, no electrospray source in the literature leverages existing electronics assembly infrastructure for component integration, giving our approach a distinct advantage at scale, although a full cost analysis is still necessary to prove economic viability. Coupled with the bulk fabrication enabled by a tethered array of 3D-printed emitters with microscale features, and the fact that 3D printing has been shown to yield cheaper per object cost in small and midbatch manufacturing,^48^ our approach may be the most fitting for easy-to-use, reliable electrospray sources for MS.

## Conclusions

This study shows that the geometric tuning of emitter geometry via additive manufacturing, coupled with control of evaporative effects and surface hydrophilicity, enables predictable increases in electrospray efficiency. The optimized emitters function well in MS protocols with a variety of solvents. Furthermore, by constraining solvent flow on an emitter, the MS signal-to-noise ratios were doubled. Also, optimization of the extractor electrode improved performance, as a higher breakdown voltage enables greater operative stability and larger peak signals. To the best of our knowledge, the surface-mounted emitters reported in this study are the first to be compatible with automated electronics assembly, eliminating issues associated with fragile capillaries or inefficiencies from alternatives, such as paper spray. The emitters are bulk fabricated in arrays via 3D printing, facilitating batch processing and scaling. High-resolution 3D-printing is ordinarily expensive for single-use parts but could enable substantial benefits at scale due to the compact size of the surface-mounted emitters.

## Supporting Information Available

Electrospray optimization, ionizer device design and fabrication, extractor air breakdown, emitter metrology and dynamics, solvent compatibility MS data, comparative MS data, and droplet movement (PDF)
