# Supplementary material for: High-Performance, Low-Cost, Additively Manufactured Electrospray Ion Sources for Mass Spectrometry
Source: J Am Soc Mass Spectrom. 2024 Mar 22;35(5):862–70. doi: 10.1021/jasms.3c00409 (PMC11066956; doi:10.1021/jasms.3c00409)
Supplement: Supplementary file 1 — js3c00409_si_001.pdf [file js3c00409_si_001.pdf]

Supporting Information

High-Performance, Low-Cost, Additively Manufactured Electrospray Ion Sources for Mass Spectrometry

Alex Kachkine[a] and Luis Fernando Velásquez-García[b,*]

[a] Department of Mechanical Engineering, Massachusetts Institute of Technology, 77 Massachusetts Avenue, Cambridge, MA, 02139, USA.
[b] Microsystems Technology Laboratories, Massachusetts Institute of Technology, 77 Massachusetts Avenue, Cambridge, MA, 02139, USA.

*Corresponding author: Luis Fernando Velásquez-García; Email: Velasquez@alum.mit.edu.

Contents

Electrospray Optimization 2

Figure S1. Electric field strength versus extractor distance and emitter tip radius. 3

Figure S2. Conical emitter model for evaporation. 4

Ionizer Device Design 6

Ionizer Fabrication 7

Table S1. Emitter Design Variants 7

Extractor Air Breakdown 9

Figure S3. Extractors and breakdown behavior. 9

Emitter Metrology 10

Table S2. Summary of Emitter Metrology 10

Figure S4. Selected SEM pictures of fabricated emitters. 10

Emitter Dynamics 11

Table S3. Observed Emitter Performance 11

Figure S5. Electrical characterzation of 3D-printed emitters. 11

Solvent Compatibility MS Data 13

Figure S6. MS spectra for evaluated solvent types. 13

Comparative MS Data 14

Figure S7. Comparative time series MS spectra for four emitter types. 14

Droplet Movement 15

Figure S8. Optical images of droplet movement on DMF device. 15

Electrospray Optimization

Emitter Electric Field. An electric field acting on the free surface of an electrically conductive liquid causes surface fluctuations and normal electrostatic forces (traction). The fluctuations locally concentrate the electric field; if the resulting traction cannot be counteracted by surface tension pulling, the instability will grow. In the threshold case of balanced forces on an equipotential surface, a stable Taylor cone with a half angle of 49.3° is generated—a consequence of Legendre polynomial solutions to the Laplace equation for an equipotential conical boundary.[1] At the apex of the Taylor cone the electric field is huge, causing charged corpuscles to be electrohydrodynamically ejected, i.e., electrosprayed. If the electrospray emitter is assumed to be hyperbolically shaped, facing a grounded planar extractor biased at a voltage V, separated a distance d, the tip electric field E_{tip} is[4]

$$E_{tip} = -\frac{2V}{R_c \ln\left(\frac{4d}{R_c}\right)} \tag{S1}$$

where R_c is the tip radius and $R_c \ll 4d$. There are two bounds of interest for the strength of the electric field acting on the emitter surface during electrospray. The lower bound is the threshold electric field required for triggering electrospray, E_{es}, which is the field at which electric traction equals the surface tension pulling, i.e.,

$$E_{es} = 2\sqrt{\frac{\gamma}{R_c \varepsilon_0}}, \tag{S2}$$

where γ is the surface tension of the liquid and ε_0 is the permittivity of free space. The upper bound is the minimum electric field needed to directly field evaporate ions, E_f, which is generally approximated as the field associated to the energy required to remove an ion from a liquid surface,[2] namely,

$$E_f = \frac{4\pi\varepsilon_0 G^2}{q^3}, \tag{S3}$$

where G is the free energy of solvation of the ions and q is the fundamental charge.

Figure S1 plots the magnitude of the electric field strength based on a parameter space relevant to atmospheric pressure MS electrospray (i.e., tip radii in the 1 nm to 1 mm range, extractor distances in the 100 µm to 1 cm range). For this parameter space, the extractor distance is significantly less influential on the field strength than the emitter tip radius, with tip radii under 10 µm necessary to trigger electrospray. While the effect of the extractor distance is more significant at distances under 100 µm, precisely achieving such distances is hard to implement as conventional positioning methods lack the required precision. Although bringing the emitter closer to the extractor can help avoid space charge-induced ion beam divergence,[12] using smaller extractor distances is not attractive for reliable MS ion sources given the manufacturing tolerances of 3D

printing (tens-of-micrometers voxels) and low-cost traditional manufacturing (a few thousands of an inch).

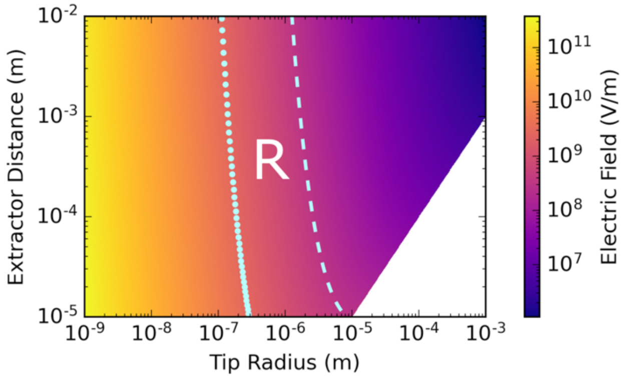

Figure S1. Electric field strength versus extractor distance and emitter tip radius. Results for an electrospray emitter biased at 2 kV ejecting isopropanol. Hyperbolic field modelling conditions cut off the plot at the lower right. Practical values are in the region marked 'R', bound by the electrospray startup field strength (dashed line) and the field evaporation startup field strength (dotted line).

Emitter Ionization Current. The current ejected by an electrospray emitter in the cone-jet mode, I_c , depends primarily on the volumetric flow rate transported by the emitter, Q, and the physical properties of the liquid[1]

$$I_c = f(\varepsilon_f)\sqrt{\frac{\gamma K Q}{\varepsilon_f}}, \tag{S4}$$

where ε_f and K are the relative electric permittivity and the electrical conductivity of the fluid, respectively, and $f(\varepsilon_f)$ is an empirical coefficient. Due to the square root dependence of (S4), the charge carried per molecule decreases with increasing flow rate; therefore, reducing the flow rate of electrospray in cone-jet mode improves the signal intensity via increased current of molecules with a high charge-to-molecule ratio. A higher ionization current, given sufficiently small length scales, can be found from field evaporation; we estimate the field evaporation current I_{FE} as[2]

$$I_{FE} = \frac{\pi R_c^2 E_{tip} k_b T}{h_p} e^{-\frac{\frac{qG}{A_v} - \sqrt{\frac{q^3 E}{4\pi\varepsilon_0}}}{k_b T}} \tag{S5}$$

where k_b is Boltzmann's constant, h_p is Planck's constant, T is the temperature of the liquid, and A_v is Avogadro's number. With organic solvents, it is expected that classical electrospray would have already jetted ions before field evaporation effects could dominate.

Emitter Evaporative Effects. We model an externally fed electrospray emitter as a solid cone with height h, tip angle θ, and base radius R_b that has a supply of fluid at the base, with flow Q_S

along the surface towards the emitter tip while undergoing uniform evaporation Q_E (Figure S2). The surface flow rate Q_S can be approximated as that of a revolved array of cylindrical capillaries scaled-down per conical surface area,[10] with the Young-Laplace pressure causing the flow, i.e.,

$$Q_S = \frac{\gamma h \pi^2 R_{cyl}^2 \cos(\theta_c) \tan(\theta)}{8\mu L \cos(\theta)}, \tag{S6}$$

where R_{cyl} is the radius of the capillary (in this case equal to the surface roughness), θ_c is the contact angle of a fluid with the emitter material, L is the length of the capillary, and μ is the dynamic viscosity of the fluid. L is modeled as the hypotenuse of the revolved cross-section of the cone as this is the distance the fluid travels to reach the emitter tip.

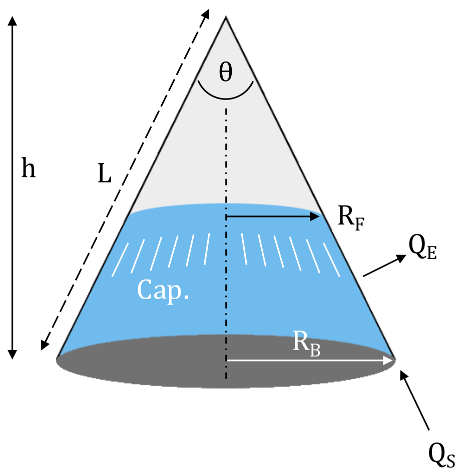

Figure S2. Conical emitter model for evaporation. The cone is formed from a revolved triangular cross-section of height h and base radius R_B, with a resulting hypotenuse L. Capillary flow along the surface is considered as a revolved array of cylindrical capillaries (*Cap.*). Fluid flows from the base with a surface mass flow rate Q_S going towards the tip while undergoing evaporation at a rate Q_E. If Q_E is larger than Q_S, the fluid will be entirely evaporated by the point it reaches a cone cross-sectional radius R_F. The fluid must reach the cone tip to be electrosprayed.

Volatile organic solvents commonly used in MS electrospray evaporate quickly at small scales as described by the Hummel-Braun-Fahrenbacher evaporation rate,[11] scaled to the surface of the emitter, i.e.,

$$Q_E = \frac{\pi r^2}{sin(\theta)} \frac{8.79 \times 10^{-5} m^{.833} P_v \left(\frac{1}{m} + \frac{1}{29}\right)^{0.25}}{T^{.05}} \sqrt{\frac{v_w}{\Delta z p_a} \frac{1}{tan\,\theta}}, \tag{S7}$$

where P_V is the vapor pressure of the liquid, m is the molecular weight of the fluid, v_w is the air speed along the fluid-gas interface, T is the absolute temperature, p_a is the absolute pressure of the gas, and Δz is the length of the fluid-gas interface, which we evaluate as the hypotenuse of the

revolved cross-section of the cone, L, for steady-state calculations. The $1/tan\,\theta$ at the end of (S7) is a normalized curvature correction needed to account for the higher amount of unsaturated air able to absorb diffusing evaporated molecules at smaller tip angles.

Evaporative Modelling Parameters. Figure 4 (main document) shows how Q_E and Q_S vary with the emitter cone semi-angle $\theta/2$ for water and isopropanol (vapor pressure equal to 2.3×10^{-2} atm and 4.3×10^{-2} atm, respectively) for a 1 mm-tall emitter, with the other parameters based on previously characterized ZnONW-coated emitters,[6] e.g., R_{cyl} is about 5 µm based on scanning electron microscope (SEM) metrology, the air speed relative to the surface of the emitter is assumed equal to 0.1 m/s (reasonable for an extractor distance of several mm and the conditions of laboratory air flow expected in our experiments), temperature (298 K), and gas pressure equal to the atmospheric pressure.

Manufacturing Tolerance Effects. We can quantify the effects of manufacturing tolerance by considering variation in the radial axis of a solid, conical emitter tip; this variation amplifies the height variation for high aspect-ratio emitters. For a differential change in radius ∂r, the change in height for a cone with angle θ is $\partial h = \partial r/tan(\theta/2)$. This effect can be significant, as the 3D printing manufacturing tolerance is on the order of at least a voxel (in general, equal to several tens of microns of side), while the height of the emitters studied in this work is on the order of 1 mm. For example, the corresponding change in height for an emitter with a tip angle of 15° for a 100 µm tolerance is around 750 µm—about the emitter height. Therefore, this change can cease electrospray or cause electric discharge via proximity to the extractor. Wider tip angles provide a more reproducible geometry, and a balance must be struck between controlling the surface flow rate and retaining consistent performance across a batch of emitters.

Extractor Modelling. Using Comsol Multiphysics 6.0 (COMSOL, Burlington, MA, USA), the effects of both emitter and extractor geometries are simulated, modeling the electric field and its relation to maximal surface strength. In the simulations, two primary geometric variations of standard cylindrical inlets are considered: (i) removal of material within a hemisphere centered around the emitter tip, and (ii) varied edge fillet radii. Three extractor designs were considered. Extractor design A is the extractor commonly used as inlet in many MS instruments— a cylindrical electrode with sharp edges (fillet radius < 50 µm). Extractor design B is a cylindrical electrode with material removed within a hemisphere with 1.5 mm radius centered around the emitter tip and a 1 mm edge fillet radius. Extractor design C is a cylindrical electrode with material removed within a hemisphere with 2 mm radius centered around the emitter tip and a 0.5 mm edge fillet radius.

Ionizer Device Design

This section covers detailed design information regarding the ionizers with surface-mounted emitters and horizontally mounted emitters explored in this study.

Ionizer with Surface-Mounted Emitter. The external casing of the device has ridges and backing planes to fully constrain the PCB and a second layer via a snap-fit connection; the second layer protects sensitive surfaces and enables phase separation of droplets. Visual features delineate the entry port and electrospray outlet. A card edge connector interfaces with controlling pads on the PCB. Voltages are applied to move droplets along the actuating electrode array, to control the electrospray emitter, and to check for correct chip insertion via an interconnect on the DMF PCB. The DMF PCB has two copper layers on opposing sides; each layer is 30 µm thick and is surfaced with 15 µm thick solder mask. There is a 300 µm separation between adjacent square droplet actuating electrodes—the minimum feature size allowed for low-cost, scalable, commercial PCB manufacturing. The copper electrodes are 50 µm thick, contributing enough surface non-uniformity to adversely affect droplet movement; this addressed by applying a 15 µm thick Kapton tape on the droplet pads to provide a smoother surface on which droplets can move. A layer of Teflon AF 2400X (DuPont, Wilmington, USA) on the dielectric layer forms the hydrophobic layer necessary for electrowetting.

Planar Ionizer. The 3D-printed ionizers are compatible with bulk fabrication: arrays of aligned ionizers can be processed to apply necessary surface coatings to the emitter, which are extended to the entire ionizer to enable capillary surface-driven flow towards the emitter. To avoid sample leakage onto the receptor, a Teflon AF 2400X dip-coat is performed on the rear of the planar ionizers. After manufacturing, the ionizers are separated from the array for individual use.

Ionizer Fabrication

This section describes the design variants and fabrication procedures for emitters, extractors, and DMF devices. The emitter variations are summarized in Table S1. For these emitters, the emitter height was set to 1.5 mm, and the tip angle was set to 15°, 30°, 60°, or 98.6° (i.e., the Taylor cone angle). For comparative purposes, we also evaluated two popular MS electrospray emitters made via traditional manufacturing: paper emitters, made via laser cutting of paper feedstock (Grade I Chromatography Paper, Bartovation, White Plains, NY, USA), and coated blades, made by stamping of sheet metal (Restek, Bellefonte, PA, USA).

The processes and materials selected to manufacture the electrospray emitters stemmed from the optimization of component bulk production; precise additive methods are inherently useful for the direct manufacture of emitter features, where critical length scales are on the order of micrometers. Emitters are made in monolithic arrays, with the specific geometry determined by the integration method (i.e., surface mount vs. horizontal mount). While high-pressure metal casting can, in theory, create emitters with tip radii on par to those made via binder jetting of SS 316L (~100 µm), the difficulty of reproducibly manufacturing such fine geometries renders the approach impractical. The same assessment is made for plastic injection molding, where reproducible high aspect ratio features at the <100 µm length scale needed for emitter fabrication are untenable.[9] Issues spanning inhomogeneous material solidification at the microscale, part warping, and mold fabrication for emitter features lead to additive approaches being far more plausible for scaling up production of micro and nanostructured electrospray emitters.

Table S1. Emitter Design Variants. Emitter types $\boldsymbol{E_1}$ and $\boldsymbol{E_2}$ correspond to novel, 3D-printed emitter designs; mainstream paper emitters and coated blades were also characterized in thi study for comparison.

Kind of manufacturing	Additive		Subtractive	Formative shaping
Fabrication process	Binder jetting		Laser cutting	Pressing
Base material	SS 316L		Grade I Chromatography Paper	Sheet metal
Identifier in study	E_1	E_2	Paper	Coated blade
Electropolishing	✓	×		
ZnONW coating	✓	✓		

Emitter Body Fabrication. Emitter designs E_1 and E_2 are made in stainless steel (SS) 316L via binder jetting. We have shown before that binder jetting of SS 316L is a robust process for making electrospray emitters, especially when post-processed with electropolishing.[3,6] Here, we expanded the approach to arrays of separable emitters. Emitter E_1 undergoes electropolishing to reduce the tip radius, as sharper tips can reduce the formation of multiple diverging electrospray jets, which could otherwise decrease signal. The electropolishing procedure involves the immersion of bare emitter tips 2 mm deep in a solution consisting of 50% phosphoric acid, 30% sulfuric acid, and

20% deionized water by volume. A positive 10 V bias voltage is applied to the part relative to a copper electrode shielded from direct access from the cathode by a glass slide. For a 5 by 4 array of metal emitters, the drawn current increases from 9 A to 10 A over the course of the electropolishing step. Electropolishing for 3 minutes removes 100 μm to 150 μm of material, reducing the emitter tip diameter to under 50 μm, in some cases resulting in <1 μm tip radii. SEM metrology of the emitters suggests that part of the spread in tip radii is due to the spacing between bound metal particles in the printed part and consequent exposure of voids during electropolishing.

Emitter Coating. Emitter designs E_1 and E_2 are coated in a conformal ZnONW film. The coating process begins with ultrasonic cleaning of the emitter in isopropanol followed by deposition of a 20 nm thick seed layer of ZnO via sputtering (AJA Orion 5 RF Sputtering system, AJA International, Inc., Scituate, MA, USA; full protocol is described in [3]). The ZnONWs are hydrothermally grown at 90° C for 1 hour via upside-down immersion in a solution consisting of equal volumetric parts of 0.025 M $Zn(NO_3)_2 \cdot 6H_2O$ and 0.025 M hexamethylenetetramine, both dissolved in deionized water. Following ZnONW growth, the parts are cleaned ultrasonically in water to remove any larger crystal clusters that protrude from the emitter surface.

Emitter Array Processing. The manufacture of the emitters harnesses the benefits of 3D printing for bulk production of finely featured components. Given that there are multiple processing steps in making the emitters, structured arrays of emitters greatly simplify the handling process. In these designs, the emitters are attached to a global frame using an array of 0.5 mm thick tethers connected to the emitter bases.

Horizontally Mounted Emitters. The horizontally mounted emitters were made via binder jetting of SS 316L using a vendor (i.Materialise, Leuven, Belgium). Emitter preparation was identical to that of surface mount devices. To avoid samples leaking onto the receptor, the ends of the planar ionizers were dipped into Teflon AF 2400X (DuPont, Wilmington, DE USA) and air-dried, forming a hydrophobic barrier. The receptor was made of SolusProto resin (Junction 3D, Santa Clarita, CA, USA) via vat photopolymerization using the digital light processing printer Asiga MAX X27 UV (Asiga, Alexandria, Australia).

Extractor Electrodes. All extractor electrode designs considered in this study (i.e., designs A, B, and C) were made via binder jetting of SS 316L using a vendor (Materialise, Leuven, Belgium).

Integrated Ionizer Assessment. The ionizers with surface-mounted emitter have an external casing that allows easy handling of the device and protects its functional components. The positioning variability, tested optically, was ~200 μm in the XY plane of the PCB, and ~50 μm in the Z direction (i.e., the vertical axis of the emitter). It was found that all emitters could be soldered onto the designated pad of the DMF PCB. Emitters could be constrained with pressure during soldering, reducing the XY positioning error to < 200 μm. This error is large enough to affect the fraction of ions that goes into the MS inlet, though the effect is mitigated by the significant airflow near the inlet of an MS instrument; most ions make it into the inlet, with experiments in the literature confirming the matter optically.[7] For planar ionizers with horizontally integrated emitters, the ionizer could be readily snapped in the bespoke holder prior to positioning.

Extractor Air Breakdown

Figure S3A shows fabrication results of 3D-printed extractors mounted to PBCs with card edge connectors. Figure S3B shows an example of an arc formed between the emitter tip and the extractor electrode—these are high-electric field channels that need to be avoided to operate the emitter as an electrospray source.

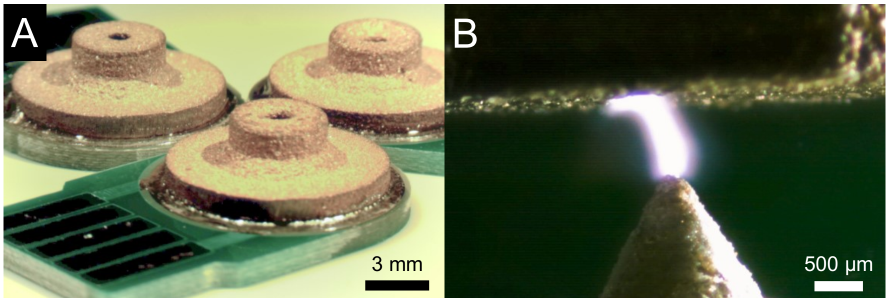

Figure S3. Extractors and breakdown behavior. A) Three fabricator extractors soldered to PCBs with card edge connectors for experimental characterization. B) Air breakdown between cylindrical extractor ***A*** and a surface mount emitter, showing an arc between tip of emitter and interior edge of extractor.

Emitter Metrology

This section reports the fabrication variation data and SEM analysis of the 3D-printed emitters, and the homogeneity of the ZnONW forests. The summary of the metrology of the emitters is in Table S2, while Figure S4 shows typical ZnONW forests grown on top of the emitters.

Table S2. Summary of Emitter Metrology. The table reports the mean (in each case, from over 60 independent measurements) and standard deviation (in parenthesis) for emitter tip angle, radius, and height. In all the emitters measured, the tip angles were nominally 30°, and all tip heights were nominally 1.5 mm. Tip radii are determined by fitting an arc to the bulk emitter material, rather than selecting for the sharpest protruding feature.

Emitter	E_1	E_2
Angle (°)	29.1 (2.54)	35.0 (2.70)
Radius (μm)	56.0 (24.6)	77.3 (39.2)
Height (mm)	1.16 (0.05)	1.26 (0.07)

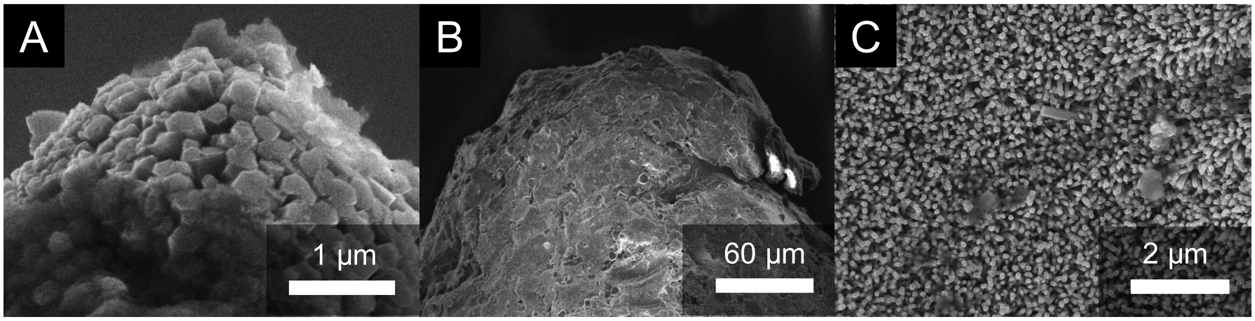

Figure S4. Selected SEM pictures of fabricated emitters. A) Closeup of a ~1 μm tip radius $\boldsymbol{E_1}$ emitter capped with short, ~150 nm diameter ZnONWs. B) Side view of the tip of an $\boldsymbol{E_2}$ emitter; surface roughness is caused by SS 316L printed particles and ZnONWs. C) Closeup of a ZnONW-coated surface.

ZnONW Forest Homogeneity. The ZnONW forests comprise normally oriented, C-oriented hexagonal crystals with heights spanning 200 nm to 2 μm, widths in the 150 nm to 250 nm range, and adjacent nanowire center-to-center separation between 300 nm and 1 μm. Metrology of ZnONWs grown on top of electropolished, 3D-printed emitters made of SS 316L via material jetting (the exact materials and processes used in this study) is reported elsewhere[3]. The growth consistency of the ZnONW forests was greatly affected by surface homogeneity. For the sharpened emitters E_1, internal voids exposed by electropolishing cause surface undulations that, in turn, negatively affect the uniformity of the ZnONW forest growth. More electropolishing can lead to a smoother surface, but it is not essential to attain a sharp emitter tip. Clumps of larger ZnONWs are sometimes present on the surface of the emitters.

Emitter Dynamics

This section presents the experimental results of current-voltage testing of emitter variants, including aggregate observed data and time-series analysis, informed by optical imaging.

Table S3. Observed Emitter Performance. Minimum tip angle for electrospray with isopropanol, maximum bias voltage before breakdown using extractor $\boldsymbol{C}$, and steady-state current for each of the emitter designs explored in this study. $\boldsymbol{E_1}$ and coated blades did not reach steady-state after electrospraying for a few tens of seconds (the current fluctuated or quickly decreased); the quantities reported here are median values. We attribute such behavior to rapid solvent depletion for $\boldsymbol{E_1}$, and poor surface fluidic control for coated blades

Emitter type	Minimum tip angle (°)	Maximum bias voltage (V)	Steady-state current (nA)
E_1	60	3000	175
E_2	30	3000	100
Paper	N/A	2750	175
Coated blade	N/A	3000	100

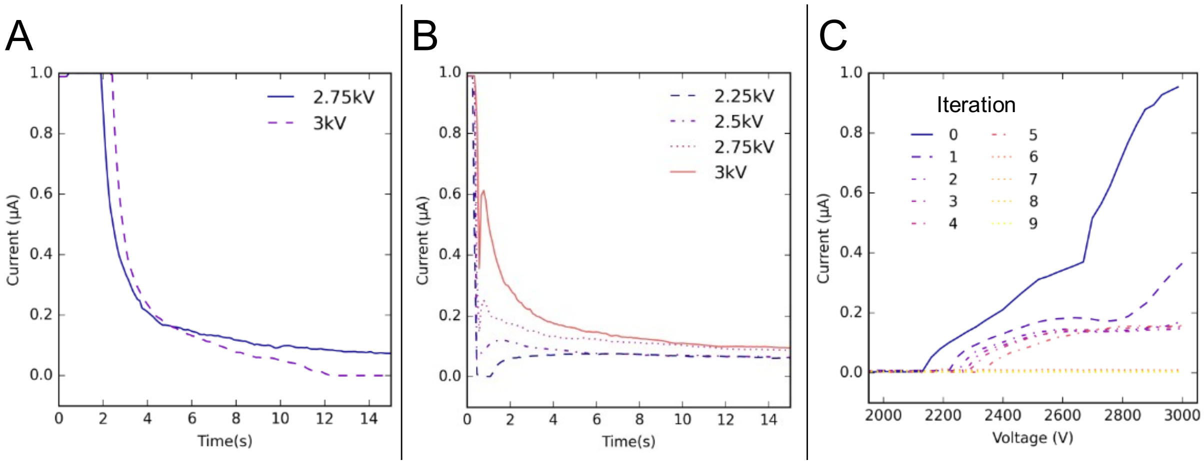

Figure S5. Electrical characterization of 3D-printed emitters. A) Emitted current versus time for two different bias voltages using an emitter $\boldsymbol{E_2}$ with a 60° tip angle. Due to the large tip angle, flow is not constrained, and bulk behavior is observed; larger bias voltages cause faster depletion of solvent. B) Emitted current versus time for four different bias voltages using an emitter $\boldsymbol{E_2}$ with a 30° tip angle. The shallower tip angle constrains the flow better, ensuring the operation of surface microscale features, while still allowing electrospray formation. Larger bias voltages lead to higher steady-state currents. C) Emitted current versus voltage for an $\boldsymbol{E_2}$ emitter with an 98.6° tip angle; 10 repetitions from 2 kV to 3 kV are plotted. The first voltage sweep reaches high currents (~1 µA

at peak); the second sweep marks the transition between startup and steady state; voltage sweeps 3-6 attain steady state, and voltage sweeps 7-10 occur after solvent has run out. This bulk behavior is tied to the dynamics of electrospray startup, with the steady-state current limited by bulk fluid behavior due to the establishment of a flow field dominated by a hydraulic impedance.[3,4,8]

Given a tip angle adequate for solvent to reach the tip of a ZnONW-coated emitter, several emission behaviors were observed depending on the other tip parameters. Emitter E_2 sometimes triggered the formation of multiple jets at the tip, spatially spread out due to charge repulsion, although still broadly directed towards the extractor. E_1 typically had a single jet that was directed towards the extractor. In some cases, the jet was not visible using the optical equipment, possibly due to immediate evaporation of the solvent at the jet nexus. Multiple jets are present in paper spray and in coated blade spray devices, which spatially diverge away from the extractor inlet due space charge effects. As a comparison, capillary electrospray can be readily made stable with appropriate electric field application, but such a method is far more difficult to integrate into a disposable consumable. Reaching the performance of our emitters would require shrinking a capillary diameter to the point where clogging and fragility become significant reliability concerns.[5]

The ionizers reported in this study only have one emitter. Nonetheless, electrical characterization of arrays (up to 85 elements in a 2-cm diameter active area) of electropolished, 3D-printed electrospray emitters made of SS 316L via material jetting, coated with hydrothermally grown ZnONWs (the exact materials and processes used in this study), showing that the emitters operate uniformly, is reported elsewhere[3].

Solvent Compatibility MS Data

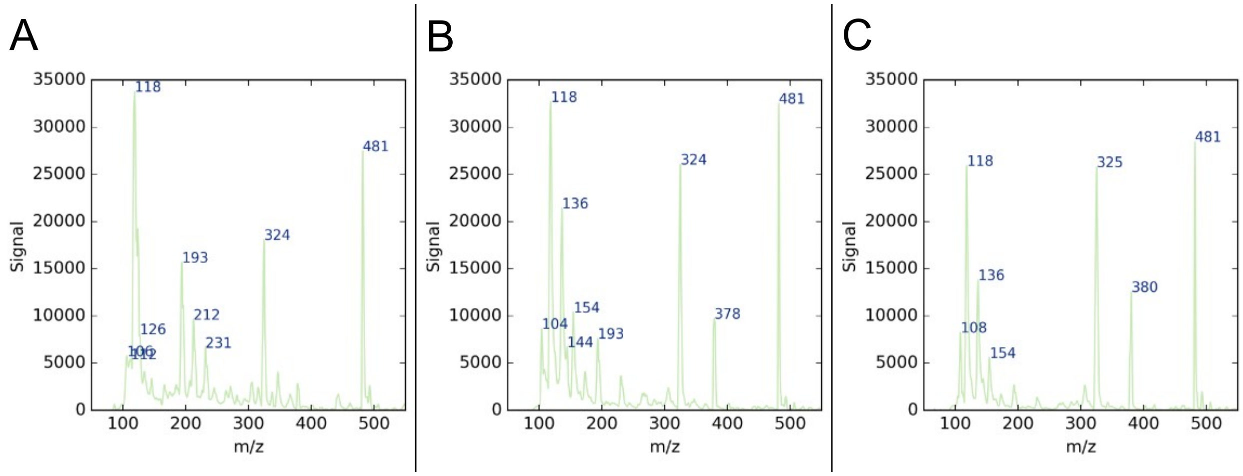

Figure S6. MS spectra for evaluated solvent types. MS peaks from 10 pharmaceutically relevant compounds at 1 µg/ml concentration, electrosprayed with an $\boldsymbol{E_1}$ emitter using A) isopropanol, B) methanol, and C) acetonitrile as solvent. Several compounds are identifiable from key peaks based on reference capillary ESI data: Nicardipine (m/z = 480), Clopidogrel (m/z = 322), Aspirin (m/z = 136), Enalapril (m/z = 378), and Rivaroxaban (m/z = 193).

Comparative MS Data

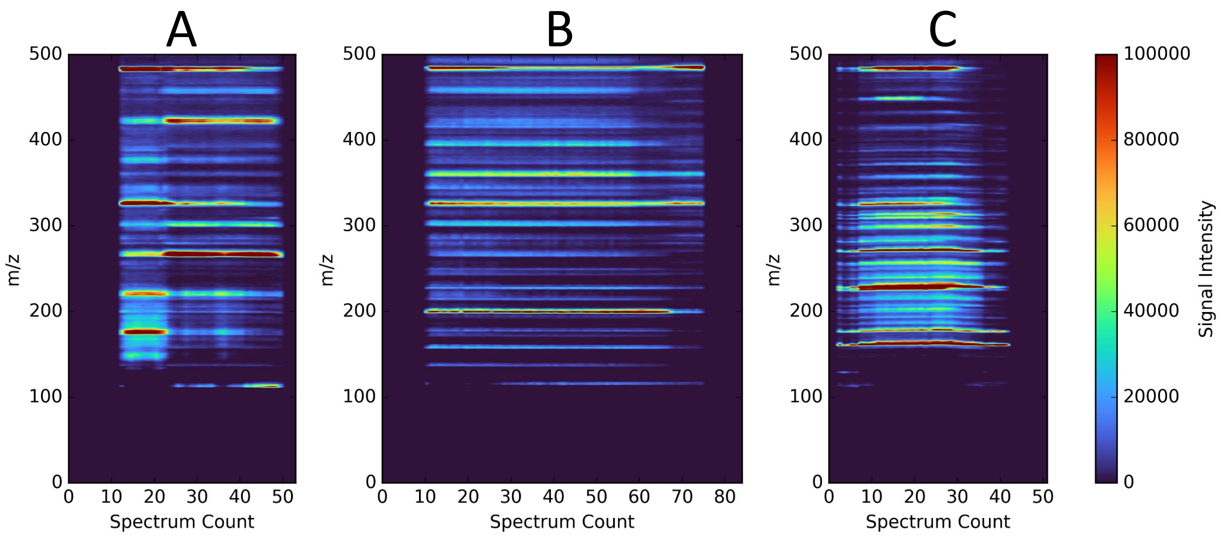

Figure S7. Comparative time series MS spectra for three emitter types. The signal intensity color scale is shared by all panels. A) Data from a ZnONW-coated emitter $\boldsymbol{E_1}$ showing high intensity signals with significant time variance. B) Data from a ZnONW-coated emitter $\boldsymbol{E_2}$ showing long stability of signals. C) Data from a mainstream Restek coated blade showing shifting signals.

Droplet Movement

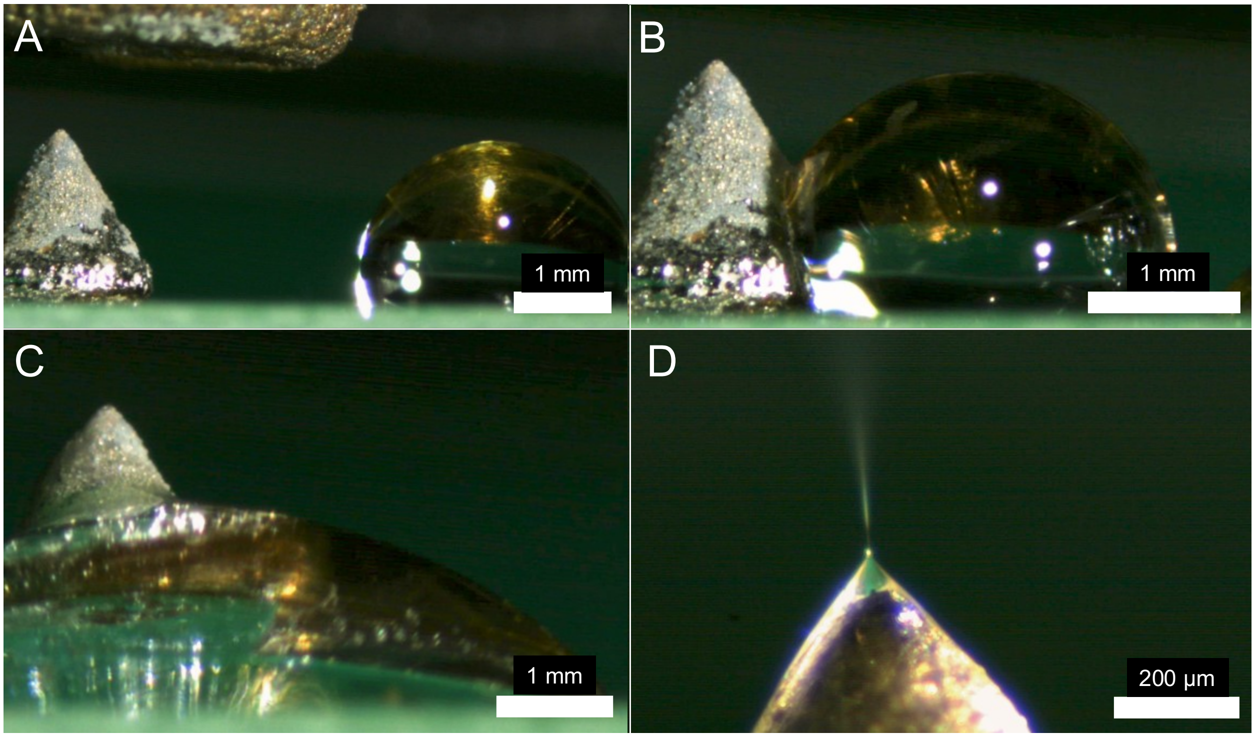

Figure S8. Optical images of droplet movement on DMF device. The 5 µL drop is 95% water, 5% acetic acid, differing in composition from other solvents used for MS analysis and likely requiring downstream protocol modifications to optimize performance. A) Drop placement on a DMF electrode. B) Droplet moved to proximity of emitter via successive application of voltages to the DMF electrodes using the card edge connector. C) Droplet starts to crawl up the hydrophilic surface of emitter after contact. D) Electrospray is initiated by biasing a voltage between the emitter and a vertically oriented extractor **C** (not in frame). Droplets moved via electrowetting are quick, switching positions in < 100 ms. The process of wetting the emitter is also quick, resulting in fast droplet processing and ionization.

References

1. Taylor, G. I. Disintegration of Water Drops in an Electric Field. *Proc. R. Soc. Lond. Ser. Math. Phys. Sci.* **1964**, *280* (1382), 383–397. https://doi.org/10.1098/rspa.1964.0151.
2. Iribarne, J. V. On the Evaporation of Small Ions from Charged Droplets. *J. Chem. Phys.* **1976**, *64* (6), 2287. https://doi.org/10.1063/1.432536.
3. Máximo, D. V. M.; Velásquez-García, L. F. Additively Manufactured Electrohydrodynamic Ionic Liquid Pure-Ion Sources for Nanosatellite Propulsion. *Addit. Manuf.* **2020**, *36*, 101719. https://doi.org/10.1016/j.addma.2020.101719.
4. Hill, F. A.; Heubel, E. V.; de Leon, P. P.; Velásquez-García, L. F. High-Throughput Ionic Liquid Ion Sources Using Arrays of Microfabricated Electrospray Emitters With Integrated Extractor Grid and Carbon Nanotube Flow Control Structures. *J. Microelectromechanical Syst.* **2014**, *23* (5), 1237–1248. https://doi.org/10.1109/JMEMS.2014.2320509.
5. Shih, S. C. C.; Yang, H.; Jebrail, M. J.; Fobel, R.; McIntosh, N.; Al-Dirbashi, O. Y.; Chakraborty, P.; Wheeler, A. R. Dried Blood Spot Analysis by Digital Microfluidics Coupled to Nanoelectrospray Ionization Mass Spectrometry. *Anal. Chem.* **2012**, *84* (8), 3731–3738. https://doi.org/10.1021/ac300305s.
6. Kachkine, A.; Velásquez-García, L. F. Nanowire-Coated Emitter Electrospray Ionizer Coupled to Digital Microfluidics For Liquid Analysis. In *2022 IEEE 35th International Conference on Micro Electro Mechanical Systems Conference (MEMS)*; **2022**; pp 95–98. https://doi.org/10.1109/MEMS51670.2022.9699691.
7. Markert, C.; Thinius, M.; Lehmann, L.; Heintz, C.; Stappert, F.; Wissdorf, W.; Kersten, H.; Benter, T.; Schneider, B. B.; Covey, T. R. Observation of Charged Droplets from Electrospray Ionization (ESI) Plumes in API Mass Spectrometers. *Anal. Bioanal. Chem.* **2021**, *413* (22), 5587–5600. https://doi.org/10.1007/s00216-021-03452-y.
8. Olvera-Trejo, D.; Velásquez-García, L. F. Additively Manufactured MEMS Multiplexed Coaxial Electrospray Sources for High-Throughput, Uniform Generation of Core–Shell Microparticles. *Lab. Chip* **2016**, *16* (21), 4121–4132. https://doi.org/10.1039/C6LC00729E.
9. Nakao, M.; Yan, C.; Yoda, M. Injection Molding Techniques for the Fabrication of MEMS Elements. In *MEMS/NEMS: Handbook Techniques and Applications*; Leondes, C. T., Ed.; Springer US: Boston, MA, **2006**; pp 870–879. https://doi.org/10.1007/0-387-25786-1_21.
10. Hassan, S.; Tariq, A.; Noreen, Z.; Donia, A.; Zaidi, S. Z. J.; Bokhari, H.; Zhang, X. Capillary-Driven Flow Microfluidics Combined with Smartphone Detection: An Emerging Tool for Point-of-Care Diagnostics. *Diagnostics* **2020**, *10* (8), 509. https://doi.org/10.3390/diagnostics10080509.
11. Hummel, A. A.; Braun, K. O.; Fehrenbacher, M. C. Evaporation of a Liquid in a Flowing Airstream. *Am. Ind. Hyg. Assoc. J.* **1996**, *57* (6), 519–525. https://doi.org/10.1080/15428119691014729.
12. Krutchinsky, A. N.; Padovan, J. C.; Cohen, H.; Chait, B. T. Maximizing Ion Transmission from Atmospheric Pressure into the Vacuum of Mass Spectrometers with a Novel Electrospray Interface. *J. Am. Soc. Mass Spectrom.* **2015**, *26* (4), 649–658. https://doi.org/10.1007/s13361-014-1062-1.
